# The Celluloid Base

**Published:** 1871-09

**Authors:** 

**Affiliations:** New York.


### ARTICLE V.

### *The Celluloid Base.*

By Preterre Brothers, New York.

We have the pleasure of communicating the result of a series of carefully conducted experiments, by which we are enabled to simplify and improve the process of moulding plates out of "The Celluloid Base," so that now it can be used for dental plates with perfect success as a substitute for rubber.

As we do not intend to take out patents for our improvements and discoveries, and wish our friends to share in the advantages they produce, we will describe the details of two processes (either of which may be used) of moulding dental plates, in which we dispense with all the usual machinery, except the flask and screws.

We prepare the flask, cut the channels and gates for the escape of the surplus material, select the plate for the base, and place it over the mould in the flask, according to the printed directions for using the celluloid base, with the exception that no gum is mixed with the plaster of which the mould is formed. Then commence our improvements; in which both of these processes the ordinary rubber flask can be used as well as the special one designed for the celluloid base. In neither of them is oil used as the medium of communicating heat to soften the material, and in both of these processes the plaster must be ordinarily moist.

In one process the flask and material, prepared as above described, are plunged into boiling water; almost as soon as the boiling water touches the material it begins to soften, and the tightening of the screws can commence, and they may be further tightened every half minute, until the edges of the flask are pressed close together, when the moulding will be completed, and the flask must be plunged in cold water to cool the case. Upon opening the flask, the plate will be found as perfectly moulded as if it had been immersed in *oil* at a temperature of three hundred degrees. We have moulded plates by this process in five minutes.

In the other process, the plaster mould in the flask must be moist, the material for the base is placed on the mould in the flask, the upper part put on, and the flask so prepared placed over a jet of gas, or the flame of an alcohol lamp; as soon as the steam begins to issue from the wet plaster, the material will be soft enough to commence tightening the screws, and in a few seconds the two parts of the flask may be brought entirely together, and the moulding of the plate completed, and the case must be cooled as already described.

In the last process, the channels and gates that are cut to receive the surplus material must be so arranged that none of it can touch the iron flask, as the flask becomes so hot that the material would ignite if brought in contact with it, and the case be spoiled. Should the material by any accident ignite, plunge it into cold water. If this is done quick enough, the case may probably be saved.

We think it a matter for congratulation, that in the different preparations of collodion that are known by the name of "rose pearl," "pyroxyline," "the celluloid base," etc., the profession can find substitutes for rubber in the making of dental plates.

So that the aims of these extortionating companies may be defeated, we shall always place before our friends any improvements that we may make that will tend to accomplish that object.—*Dental Cosmos.*

---

